# Intention to Screen for Alzheimer’s Disease by Residential Locale

**DOI:** 10.3390/ijerph17072261

**Published:** 2020-03-27

**Authors:** Lilah M. Besser, Willa D. Brenowitz, Juyoung Park, Magdalena I. Tolea, James E. Galvin

**Affiliations:** 1 School of Urban and Regional Planning, Institute for Human Health and Disease Intervention, Florida Atlantic University, Boca Raton, FL 33431, USA; 2 Department of Psychiatry, School of Medicine, University of California San Francisco, San Francisco, CA 94158, USA; Willa.Brenowitz@ucsf.edu; 3 Phyllis and Harvey Sandler School of Social Work, Florida Atlantic University, Boca Raton, FL 33431, USA; jpark14@fau.edu; 4 Comprehensive Center for Brain Health, Department of Neurology, Miller School of Medicine, University of Miami, Miami, FL 33136, USA; mit38@med.miami.edu (M.I.T.); jeg200@miami.edu (J.E.G.)

**Keywords:** Alzheimer’s disease, dementia, screening, intention, neighborhood, urban, rural, suburban

## Abstract

A random digit dialing sample from Missouri (USA) was used to compare associations between psychosocial factors and Alzheimer’s disease (AD) screening intention based on residential locale. Linear regression associations between demographics and five psychosocial constructs (dementia knowledge, perceived screening benefits, preventive health behaviors, perceived susceptibility, and self-efficacy) and screening intention were compared by residential locale. Participants (*n* = 932) had a mean age of 62 years (urban: *n* = 375; suburban: *n* = 319, rural: *n* = 238). African Americans more often lived in urban than suburban/rural neighborhoods, and more urban than suburban/rural residents reported insufficient income. Preventative health behaviors (e.g., dentist visits) were higher in urban and suburban versus rural participants. AD screening intention did not differ by residential locale. Among urban participants, self-efficacy to get screened was associated with screening intention. Among rural participants, dementia knowledge was associated with screening intention. Perceived screening benefits and perceived susceptibility to AD were associated with screening intention regardless of locale. Unlike urban participants, rural participants demonstrated greater screening intention with greater dementia knowledge. Our findings suggest that psychosocial factors associated with AD screening intention differ depending on residential locale. Strategies to increase dementia screening may need to account for regional variations to be maximally effective.

## 1. Introduction

Alzheimer’s disease and related dementias (ADRD) are expected to increase in prevalence over the coming decades, affecting approximately 88 million worldwide by 2050 [1]. Cognitive assessments remain one of the primary means by which individuals are diagnosed with ADRD in community settings. However, much remains unknown regarding how many older adults receive routine cognitive assessments and the factors predicting whether an individual will request an assessment from their physician or healthcare provider.

In 2018, the Alzheimer’s Association polled individuals ≥ 65 years old and found that only half reported ever receiving a cognitive assessment by their physician [1]. In addition, only 16% reported receiving regular cognitive assessments. In stark contrast, ≥73% of respondents reported regular assessments of their blood pressure, cholesterol levels, and hearing function. As part of the study, a separate survey was completed by 1000 primary care physicians (PCP), and the data from these two surveys revealed that expectations for cognitive assessments were poorly aligned between patients and PCPs. While most of the consumer survey respondents desired that their physicians recommend cognitive assessments, PCPs were more often relying on the patients or caregivers to request the cognitive assessments. 

Although some of this disconnect may be the result of a lack of training on how to conduct cognitive assessments, how often they should be done, and how to make a diagnosis [2,3], PCPs may also be hesitant if there is no perceived benefit to the early detection of dementia, as there are currently no medications to stop or delay the pathology that leads to ADRD [4]. However, medications are available that may help to treat cognitive, motor, affective, and behavioral symptoms [4,5].

The value of early screening and detection of cognitive impairment includes receiving drug treatments to control symptoms at earlier disease stages, as mentioned above. Early screening also would allow patients, family members, and caregivers time to adjust to the disease and plan for the future. The burden of disease to caregivers [6,7,8] and patients is a significant consideration when evaluating the benefits of early disease detection. While current drug options offer little in the way of hope of ultimately delaying disease progression, early detection to reduce the burden associated with time to diagnosis [9,10] could substantially improve quality of life for patients and their caregivers. The period of uncertainty and social and financial strains in obtaining a diagnosis is an underappreciated aspect of the burden of ADRD [10]. In addition, early detection is particularly important for identifying individuals most suitable for clinical trials for the secondary and tertiary prevention of ADRD and for treatment with any newly developed therapies coming to market in the future.

Until healthcare providers routinely incorporate cognitive screening into the annual check-up for older adults (e.g., via the adoption of consensus guidelines), the onus falls partially to patients and family members to request screening from providers. Few studies have investigated dementia screening, particularly the intention to obtain dementia screening, among older adults, and the factors that predict greater screening intention. A study in Japan found that the knowledge of preventive lifestyle factors and understanding of the seriousness of AD were associated with increased screening intention [11]. One of the few other known studies to examine this topic found that among a population-based sample in Missouri (USA), dementia screening intention was higher among those with a greater knowledge of dementia, a greater perceived benefit of receiving diagnosis and treatment, greater self-efficacy, and a greater perceived susceptibility to AD, as well as among individuals practicing more preventive health behaviors [12]. The primary goal of the Missouri study [12] was to determine the psychosocial constructs associated with screening intention, irrespective of individual-level characteristics. In that study, the Health Belief Model [12,13,14], the Theory of Reasoned Action/Planned Behavior [15,16], and Bandura’s Theory of Self-Efficacy [17] were used to test a number of predisposing factors (e.g., knowledge and attitudes), enabling factors (e.g., subjective norms), and need factors (e.g., the perceived severity of AD) hypothesized to predict AD screening intention.

The current study expands upon the previous Missouri study [12], using data from the same cohort to examine if the psychosocial predictors of intention to screen for Alzheimer’s disease (AD) differ by residential locale (urban, suburban, or rural). It is hypothesized that screening intention varies by residential setting, which captures otherwise unmeasured regional, cultural, and educational differences between individuals. The screening rates for serious health conditions such as cancer and human immunodeficiency virus are significantly lower among individuals living in rural areas compared to those in suburban/urban areas [18,19]. Although no known studies have been published, it seems likely that these geographic disparities extend to dementia screening. Strategies and interventions to increase dementia screening intention and the receipt of screening will need to account for individual and regional variations to be maximally effective. In addition, it is important to increase awareness for potential differences by residential locale, particularly because individuals living in rural environments have less access to health care, specialists, and community resources, and often have higher incidence and prevalence rates of ADRD [20,21].

## 2. Materials and Methods

In 2005, a population-based sample of 1039 individuals from three geographic areas in Missouri (USA) were recruited via random digit dialing (RDD) [12,13]. Commercial software was employed by the University of Missouri’s Center for Health Care Quality to generate the phone numbers and the computer-assisted telephone interview. The geographic areas surveyed were chosen to be inclusive of urban (St. Louis City, MO, USA), urban/suburban (St. Louis County, MO, USA), and rural residents (Adair County), to cover a range of socioeconomic classes and allow for an equitable representation of African Americans and women consistent with the underlying population. To determine eligibility, the first age-eligible, English-speaking household adult (>50 years) completed the Short Blessed test [22], and those scoring < 6 (i.e., with no dementia) were asked to complete the interview. Additional details about the RDD methods are available elsewhere [12].

The primary aim of the RDD study was to assess knowledge and beliefs about AD and memory loss, and about the availability and access to screening tests. The questionnaire was developed to survey multiple psychosocial constructs that may predict AD screening intention.

### 2.1. Eligibility Criteria

In the current study, individuals who were missing data on the intention to be screened (“I plan to have a screening test for memory loss at some point in my life”) were excluded, as were those missing data on residential locale (urban, suburban, or rural).

### 2.2. Survey Data

Demographics collected included age, sex, race (white, black/African American, Asian, Native Hawaiian/other Pacific Islander, American Indian/Alaska Native, other), Hispanic ethnicity, education (<7 years, junior high, partial high school, high school graduate, partial college, college graduate, graduate professional training), income, and marital status. Health-related characteristics that were assessed include family history of AD, and self-rated mental health and physical health (poor, fair, neutral, good, excellent).

A number of psychosocial assessments were administered during the telephone interview, based on both pre-existing and new questions designed for the RDD study. The survey and the conceptual model linking the psychosocial factors and AD screen intention has been validated and described previously [13]. Briefly, the psychosocial constructs originally hypothesized to be associated with screening intention included knowledge/attitudes about AD, the perceived benefits to being screened, the perceived barriers to being screened, psychological traits (e.g., anxiety and life orientation), social support/networks, subjective norms (e.g., if friends/family would support getting screened), the perceived availability/accessibility of screening services, the perceived severity of the disease, perceived susceptibility to the disease, and perceived mental and physical health status.

In a subsequent study after the RDD questionnaire was validated, the strongest psychosocial predictors of AD screening intention were identified as (1) dementia knowledge (e.g., knowledge of the associated financial burden), (2) perceived benefits of screening (e.g., early diagnosis allows for planning), (3) preventive health behaviors (e.g., dentist visits), (4) perceived susceptibility (e.g., understanding that with age, more likely to get AD), and (5) self-efficacy to be screened (e.g., confident that can get a screening test for memory loss) [12]. Within each of these latent constructs, the authors of the previous studies identified key questions (Table 1) that were most strongly associated with those constructs, and those questions were used in this study to create index measures of the five constructs predicting screening intention. Each question was assessed on a 10-point scale, where 1 = strongly disagree and 10 = strongly agree. As each construct was strongly predicted by a varying number of questions, the value of each construct was determined by a simple average of scores for each question falling under a given construct. Therefore, the variables for each construct are measured on a scale of 1 to 10, where higher scores equate to greater dementia knowledge, perceived benefits of screening, preventive health behaviors, perceived susceptibility to AD, and self-efficacy to be screened.

The intention to screen for AD was assessed with the question, measured on a scale of 1 = strongly disagree to 10 = strongly agree, “I plan to have a screening test for memory loss at some point in my life”.

### 2.3. Variables and Statistical Analyses

Demographics, family history of AD, self-reported mental and physical health (good/excellent versus poor/fair/neutral), Short Blessed Test scores (higher score: worse cognition), psychosocial construct scores, and AD screening intention are described for the sample using means and standard deviations (SD) or frequencies and percentages. The demographics included age, sex, Hispanic ethnicity, race (white, African American, or other), education (less than high school, high school, or college or higher), income (just or not enough to make ends meet, versus comfortable), and married/partner status (versus widowed/single). Descriptive statistics (chi-square/exact tests or analysis of variance) were used to test for significant differences in these characteristics according to residential locale (urban, suburban, or rural). For characteristics that differed by residential locale using the omnibus tests, post-hoc contrasts (Tukey’s Studentized Range Test or unadjusted logistic regression using Bonferroni-corrected *p*-values) were run to test for differences between specific categories of participant characteristics.

Linear regression models, stratified by residential locale, were run to examine whether bivariate associations between participant characteristics, psychosocial constructs, and AD screening intention differed among urban, suburban, and rural participants. Any characteristics associated with AD screening intention at *p* < 0.20 in the bivariate models were included in multivariable models. The multivariable models were stratified by urban, suburban, and rural residence, and significant differences in associations with psychosocial factors by residential locale were tested using interaction terms (e.g., dementia knowledge × residential locale, where locale was entered as two dummy variables: suburban versus urban, rural versus urban). The interaction models controlled for covariates and the main effects that composed each interaction term. Statistical significance for the multivariable models was based on an alpha = 0.05 (associations with *p* < 0.01 and *p* < 0.001 are noted in tables to allow for multiple comparisons).

## 3. Results

**Figure 1:**
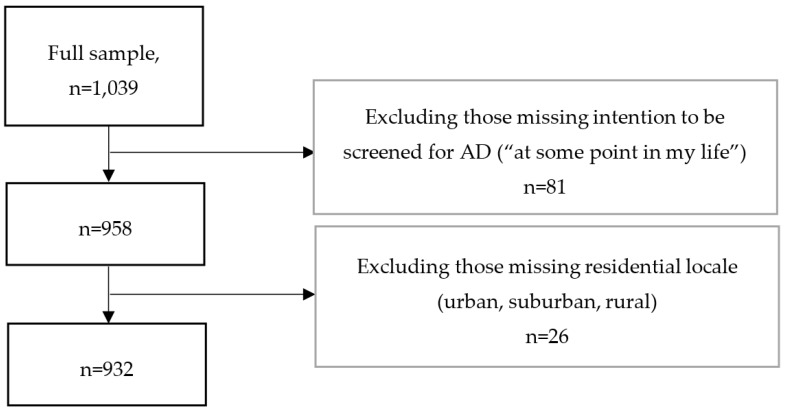
Sample size flow chart.

The resulting analytic sample included 932 participants (Figure 1), comprised of 375 urban, 319 suburban, and 238 rural residents (Table 2). The mean age was 62 years (SD = 10), 67% were female, 86% were white, 1.4% were Hispanic, and the majority had at least some college education (67%) (Table 2). Participants living in urban and suburban areas were on average a few years older (63 years) than rural residents (61 years), with no differences in sex, Hispanic ethnicity, or education level by residential locale. Twelve percent were African Americans, with the majority being of white race (86%), and there were significantly more African American participants living in urban (19%) and suburban locales (11%) versus rural locales (3%). This is consistent with the census demographics of Missouri at the time of the survey. The majority of the sample had enough income to at least make ends meet (93%), but a relatively higher percentage of those living in urban areas (9%) reported insufficient income compared to rural residents (4%). Almost half of the sample was married, but a larger percentage of those living in suburban (53%) or rural areas (60%) were married compared to those in urban areas (38%).

Approximately 25% of the participants reported a family history of AD, which did not differ by residential locale (Table 2). Good or excellent self-rated physical and mental health was reported among 70% and 85% of participants, respectively, with no differences by residential locale. Participants indicated a moderate screening intention at a certain point in their lives (mean = 5.5; SD = 3.4), which did not differ by residential locale (*p* = 0.36). Short Blessed test scores were higher among urban (mean = 1.8) compared to suburban residents (mean = 1.4).

Mean scores for the psychosocial constructs were 7.1 (SD = 3.2) for dementia knowledge, 8.0 (SD = 2.1) for perceived benefits to screening, 6.9 (SD = 2.7) for preventive health behaviors, 4.6 (SD = 2.4) for perceived susceptibility, and 8.0 (SD = 2.3) for self-efficacy to get screened (Table 3). Participants living in urban areas had greater dementia knowledge than those in suburban areas, and individuals in suburban areas self-reported more preventive health behaviors than those living in urban and rural areas. 

In the unadjusted models, age, sex, race, education, income, and family history of AD were associated with AD screening intention at *p* < 0.20 for at least one of the residential locale groups (urban, suburban, or rural) and were therefore included in the multivariable models (Table 4). All five psychosocial constructs were associated with screening intention at *p* < 0.05, but some associations varied by residential locale. Dementia knowledge was a significant predictor (i.e., *p* < 0.05) only among rural residents, preventive health behavior was a significant predictor only among urban residents, and self-efficacy to get screened was a significant predictor only among urban residents.

In the adjusted analyses, increasing age was associated with a lower intention to be screened for AD among urban and suburban but not rural residents (Table 5). Males living in suburban areas had a lower intention to be screened, associations not observed for those living in urban or rural locales. Individuals of non-white race (in this sample, primarily African Americans) and having at least some college education had a higher intention to be screened if they lived in suburban areas, an association not observed among urban and rural residents. Individuals with a family history of AD living in rural areas had a greater intention to be screened, an association not observed among urban and suburban residents.

In the adjusted models, only amongst rural participants was dementia knowledge associated with a greater intention to be screened for AD, and this was a significant difference from urban residents (interaction term *p* = 0.03) (Table 5). Having a perceived benefit of screening and perceived susceptibility to AD was associated with a greater intention to be screened among urban, suburban, and rural participants, with no differences in associations by residential locale (interaction terms, *p* > 0.05). No significant differences were observed based on residential locale (i.e., testing interaction terms) when examining associations between intention to be screened and either preventive health behaviors or self-efficacy to be screened (interaction terms, *p* > 0.05). However, when stratifying the multivariable models by residential locale, the association between self-efficacy and screening intention was significant among rural, but not suburban/rural, residents.

## 4. Discussion

Intention to be screened for AD did not vary by residential locale. However, the psychosocial constructs previously associated with intention to be screened for AD were found to vary for urban, suburban, and rural participants. Dementia knowledge was only a significant predictor of intention to be screened among rural residents, and self-efficacy was only a significant predictor among urban residents. Urban, suburban, and rural participants all demonstrated an association between a greater perceived benefit of screening and greater perceived susceptibility to AD, and greater intention to be screened. In addition, participant demographics were differentially associated with AD screening intention depending on residential locale. 

Dementia knowledge was rated lower among suburban versus urban and rural residents. In multivariable analyses, this dementia knowledge was only a significant and positive predictor of AD screening intention among rural residents. Compared to individuals living in urban and suburban areas, increased knowledge about the burden of AD may increase intention to be screened among rural residents, who can be more physically and socially isolated from friends and family, medical care, and support services. Therefore, these findings can be useful to help inform future efforts targeted at increasing dementia knowledge that may also serve to promote screening among rural residents.

The perceived benefits of screening and perceived susceptibility to AD were significant positive predictors of AD screening intention regardless of residential locale. This indicates that these psychosocial constructs may be relatively fixed predictors of screening intention irrespective of socioeconomic or cultural circumstances. Interventions and educational programs to increase awareness of these factors may have the benefit of increasing intention to be screened for AD across a larger variety of individuals, although other studies will be needed to confirm our results in different cohorts. In addition, the positive and negative consequences of influences on an individual’s perceived susceptibility to AD need to be investigated further to confirm that any interventions or programs to address that psychosocial construct will not have unintended consequences.

Self-efficacy to get screened was associated with a greater intention to obtain screening among urban residents, but not among suburban or rural residents. It is unclear why greater self-efficacy was only associated with greater screening intent among urban participants. Perhaps a greater self-efficacy to be screened makes the most difference towards intention to be screened in locations that have increased densities of medical services, such that individuals who recognize their capability of obtaining screening also have a greater intent to receive screening, given the ease with which they can access screening services in their local area. However, it must be noted that this observed association among urban, but not suburban or rural, residents was not statistically significant when tested via an interaction term in the multivariable model. Therefore, although this study is suggestive of a difference in this association by residential locale, other studies would be needed to confirm these findings.

The participant demographics associated with intention to be screened for AD differed by residential locale. Increasing age was associated with a lower intention to be screened among urban and suburban, but not rural, residents. Although this seems counterintuitive, it is possible that this observed association results from underlying differences in the awareness and knowledge of AD, which differ by age cohort and residential locale [23]. Self-rated understanding of the financial burden that AD may place on family and the perceived cost of a memory screening test was higher among older urban and suburban residents (versus younger residents) (data not shown), which may indicate that with increased awareness of the potential burdens comes an increased hesitance in receiving a screening, particularly if individuals know that there are no effective treatments. It is also possible that perceptions of screening costs and benefits change with age, irrespective of dementia knowledge or education level, and this should be further studied and accounted for in any efforts to increase screening for AD among older adults.

Male sex, non-white race, and possessing a college education were associated with screening intention only amongst suburban residents. The negative association between male sex and screening intention found only among those living in suburban areas may be partially explained by sex-differences in preventive health behaviors. Males reported lower levels of preventive behaviors than females, and these differences were primarily observed in suburban areas and not in urban or rural areas (data not shown). In addition, positive associations were observed between non-white race (primarily African Americans) and at least a college education, and greater screening intention among suburban residents, associations not observed among urban or rural residents. Differences in education level, socioeconomic status, and knowledge of AD may explain these findings, but additional studies focused on these demographic differences in screening intention by region are needed.

This study has some limitations. The surveyed geographic area is primarily composed of whites and African Americans, with few Hispanics or other typically underrepresented groups. In addition, the physical and sociocultural environments encompassed by the urban, suburban, and rural areas surveyed may not be generalizable to other areas of the US. Although the psychosocial constructs were validated, it is possible that other constructs not measured by the RDD study are important to understanding differences in AD screening intention by residential locale. While intention to be screened is a plausible predictor of actual AD screening behavior, few studies have examined this, and therefore, the results from this study cannot be interpreted as psychosocial predictors associated with obtaining cognitive screening. However, intention to obtain screening is a useful surrogate given that the majority of the population is not screened (actual behavior is rare) and intention is the single largest predictor of future behavior [15,16]. Lastly, the survey was conducted in 2005. Since that time, the public’s understanding of ADRD and intention to screen for AD may have changed. There are no known recently conducted surveys collecting similar data on the psychosocial predictors of AD screening intention. Thus, new surveys are needed, including those targeting larger geographic areas (e.g., national US surveys) as well as developing regions, which have been understudied compared to developed regions with respect to dementia screening and intention to be screened. Research is needed to understand the differences in dementia screening intention between individuals in developing and developed regions, and to understand the predictors of screening intention—and their potential variation by residential locale—within developing regions.

The strengths of the study are the use of survey data that have been validated and that are strongly rooted in models of health behavior, including the Health Belief Model [12,13,14], the Theory of Reasoned Action/Planned Behavior [15,16], and Bandura’s Theory of Self-Efficacy [17]. Additional strengths include the random sampling method employed, the high survey completer rate (72%), and the sample’s representative mix of gender, race, and geographic location that is comparable to the census demographics of Missouri at the time of the study.

## 5. Conclusions

Our results suggest that the predictors of screening intention for AD vary depending on whether an individual resides in an urban, suburban, or rural area. This study focused on Missouri residents, and thus the findings need to be replicated in other geographic regions of the US and internationally. In addition, new population-based studies are required to shed light on whether dementia screening intention and its predictors have changed in the last decade alongside increasing public awareness of Alzheimer’s disease and related dementias. If our results are replicated in future studies, the findings suggest that any strategies employed to increase dementia screening will need to be tailored to account for differences in screening intention by residential locale. Programs and interventions that target the perceived benefits of screening and susceptibility to AD may be effective regardless of residential locale, whereas increasing self-efficacy to be screened and increasing dementia knowledge may only be effective among urban and rural residents, respectively. Strategies that recognize these differences in the psychosocial predictors of screening by residential locale may be maximally effective when implemented in tandem with programs and policies to reduce disparities in access to dementia screening services.

## Figures and Tables

**Table 1:** Survey questions used to construct the five psychosocial constructs.

Construct	Questions (Scale of 1 = Strongly Disagree to 10 = Strongly Agree)
Knowledge	If the screening test for memory loss showed that my risk is high, it might have a large financial burden on me and my family.
Perceived benefits	Diagnosing memory loss at a very mild stage will allow me to get medication to treat it.
	Early diagnosis will allow me to plan my life.
Preventive health behaviors	I have regular mammograms (if female) or prostate checks (if male).
	I have regular colonoscopy examinations.
	I see the dentist regularly.
Perceived susceptibility	Compared to other people my age, I have a pretty good chance of getting Alzheimer’s disease.
	As I age, I am more likely to get Alzheimer’s disease.
Self-efficacy	I am confident I can get a screening test for memory loss.
	I am confident I could find out about how to get a screening test for memory loss.
	I am confident I can ask my doctor for a referral to get a screening test for memory loss.

**Table 2:** Participant demographics by residential locale.

Participant Characteristics ^a^	Total N = 932	Urban N = 375	Suburban N = 319	Rural N = 238	p-Value ^b^
Age, mean (SD)	62.4 (10.0)	63.0 (10.3)	63.0 (9.7)	60.8 (9.6)	0.01 ^c^
Female, n (%)	624 (67.0%)	248 (66.1%)	219 (68.7%)	157 (66.0%)	0.73
Hispanic, n (%)	13 (1.4%)	3 (0.8%)	8 (2.5%)	2 (0.8%)	0.16
Race, n (%)					
White	803 (86.2%)	295 (78.7%)	280 (87.8%)	228 (95.8%)	<0.0001 ^d^
African American	112 (12.0%)	71 (18.9%)	35 (11.0%)	6 (2.5%)	
Other	17 (1.8%)	9 (2.4%)	4 (1.3%)	4 (1.7%)	
Education, n (%)					
Less than high school	51 (5.5%)	24 (6.4%)	10 (3.1%)	17 (7.2%)	0.18
High school	254 (27.3%)	104 (27.7%)	83 (26.0%)	67 (28.3%)	
College or higher	626 (67.2%)	247 (65.9%)	226 (70.9%)	153 (64.6%)	
Income, n (%)					
Comfortable	580 (62.5%)	207 (55.4%)	221 (69.7%)	152 (64.1%)	0.001 ^d^
Just enough to make ends meet	290 (31.3%)	135 (36.1%)	80 (25.2%)	75 (31.7%)	
Not enough to make ends meet	58 (6.3%)	32 (8.6%)	16 (5.1%)	10 (4.2%)	
Married/partner, n (%)	454 (48.7%)	141 (37.6%)	170 (53.3%)	143 (60.1%)	<0.001 ^d^
Family history of AD, n (%)	226 (24.8%)	99 (26.9%)	73 (23.6%)	54 (23.2%)	0.49
Good/excellent self-rated physical health, n (%)	651 (69.9%)	250 (66.7%)	230 (72.1%)	171 (72.2%)	0.21
Good/excellent self-rated mental health, n (%)	787 (84.6%)	315 (84.2%)	272 (85.3%)	200 (84.4%)	0.92
Intention to be screened for AD, mean (SD)	5.5 (3.4)	5.7 (3.4)	5.5 (3.4)	5.3 (3.5)	0.36
Short Blessed score, mean (SD)	1.6 (2.2)	1.8 (2.3)	1.4 (2.1)	1.7 (2.2)	0.04 ^c^

Abbreviations: AD = Alzheimer’s disease; SD = standard deviation ^a^ Missing data: age, *n* = 4; Hispanic, *n* = 1; education, *n* = 1; family history, *n* = 22; physical health, *n* = 1; mental health, *n* = 2; income, *n* = 4; intention to screen in next year, *n* = 247; intention to screen after turn certain age, *n* = 258; intention to screen if have symptoms, *n* = 2. ^b^ Using chi-square tests (Fisher’s exact if appropriate) or analysis of variance ^c^ Tukey’s Studentized Range Test: differences in age when comparing urban versus rural and suburban versus rural participants and in Short Blessed Score when comparing urban versus suburban participants ^d^ Unadjusted logistic regression (Bonferroni-adjusted *p*-value): differences by race (African American and white) when comparing urban versus suburban, urban versus rural, and suburban versus rural participants; by income (comfortable versus just enough) when comparing urban versus suburban participants; and by marital status when comparing urban versus suburban and urban versus rural participants.

**Table 3:** Psychosocial predictors of intention to be screened for Alzheimer’s disease by residential locale.

Construct ^a,b^	Mean Score (SD)				*p*-value ^c^
	Total	Urban	Suburban	Rural	
Dementia knowledge	7.1 (3.2)	7.3 (3.0)	6.7 (3.2)	7.1 (3.3)	0.04 ^d^
Perceived benefits to screening	8.0 (2.1)	8.0 (2.1)	7.9 (2.2)	8.1 (2.0)	0.52
Preventive health behaviors	6.9 (2.7)	6.8 (2.7)	7.3 (2.6)	6.3 (2.6)	< 0.0001 ^d^
Perceived susceptibility	4.6 (2.4)	4.6 (2.5)	4.6 (2.4)	4.7 (2.4)	0.68
Self-efficacy to get screening	8.0 (2.3)	7.9 (2.4)	8.0 (2.4)	8.3 (2.2)	0.11

^a^ Missing data: Dementia knowledge, *n* = 6; Perceived benefits to screening, *n* = 20; Preventive health behaviors, *n* = 36; susceptibility, *n* = 19; self-efficacy, *n* = 9 ^b^ higher scores equate to greater dementia knowledge, perceived benefits, etc. ^c^ analysis of variance ^d^ Tukey’s Studentized Range Test: differences in dementia knowledge comparing urban versus suburban participants and in preventive health behaviors comparing urban versus suburban and suburban versus rural participants.

**Table 4:** Unadjusted associations with intention to be screened for Alzheimer’s disease by residential locale.

Characteristics	Unadjusted Estimate (b) (95% Confidence Interval)		
	Urban	Suburban	Rural
Age	−0.06 (−0.09, −0.02) ***	−0.08 (−0.12, −0.04) ***	−0.02 (−0.06, 0.03)
Male sex	−0.60 (−1.33, 0.12)	−1.10 (−1.89, −0.30) **	−0.49 (−1.43, 0.44)
Non-white race	1.17 (0.34, 2.01) **	1.23 (0.10, 2.37) *	0.45 (−1.76, 2.67)
Married/partner	−0.25 (−0.96, 0.46)	−0.25 (−1.00, 0.50)	0.21 (−0.70, 1.12)
Education			
HS vs. <HS	−2.47 (−3.97, −0.98) **	0.95 (−1.28, 3.18)	−0.43 (−2.29, 1.44)
College vs. <HS	−1.62 (−3.03, −0.20) *	1.39 (−0.76, 3.54)	−0.07 (−1.83, 1.68)
Income			
Just enough vs. comfortable	0.32 (−0.42, 1.06)	0.21 (−0.67, 1.08)	0.14 (−0.83, 1.11)
Not enough vs. comfortable	0.36 (−0.28, 0.99)	0.22 (−0.65, 1.08)	0.61 (−0.51, 1.74)
Family hx of AD	1.24 (0.46, 2.02) **	1.61 (0.74, 2.49) ***	1.75 (0.70, 2.80) **
Self-rated physical health ^a^	−0.12 (−0.85, 0.62)	0.21 (−0.62, 1.04)	0.57 (−0.42, 1.57)
Self-rated mental health ^a^	0.01 (−0.94, 0.96)	0.08 (−0.98, 1.13)	−0.78 (−2.01, 0.45)
Dementia knowledge	0.10 (−0.02, 0.21)	0.11 (−0.01, 0.22)	0.24 (0.11, 0.38) ***
Perceived benefit of screening	0.54 (0.38, 0.70) ***	0.46 (0.30, 0.63) ***	0.34 (0.11, 0.56) **
Preventive health behaviors	0.15 (0.02, 0.28) *	0.06 (−0.09, 0.21)	0.15 (−0.02, 0.32)
Perceived susceptibility	0.36 (0.23, 0.50) ***	0.17 (0.02, 0.33) *	0.38 (0.19, 0.56) ***
Self-efficacy to get screening	0.29 (0.15, 0.44) ***	0.11 (−0.05, 0.26)	0.19 (−0.01, 0.39)

Abbreviations: HS = high school; AD = Alzheimer’s disease * *p* < 0.05; ** *p* < 0.01, *** *p* < 0.001 ^a^ Good/excellent versus poor/fair/neutral.

**Table 5:** Adjusted associations with intention to be screened for Alzheimer’s disease by residential locale.

Characteristics	Adjusted Estimate (b) (95% Confidence Interval)		
	Urban	Suburban	Rural
Age	−0.05 (−0.08, −0.01) **	−0.05 (0.10, −0.01) **	0.01 (−0.04, 0.07)
Male sex	−0.24 (−0.91, 0.44)	−1.17 (−2.00, −0.35) **	−0.39 (−1.43, 0.65)
Race (non-white vs. white)	−0.73 (−0.06, 1.53)	1.38 (0.23, 2.53) *	0.06 (−2.25, 2.38)
Education			
HS vs. <HS	−1.97 (−3.44, −0.50)	1.90 (−0.26, 4.06)	−0.44 (−2.50, 1.62)
College vs. <HS	−1.14 (−2.56, 0.29)	2.23 (0.12, 4.33) *	0.01 (−2.05, 2.06)
Income			
Just enough vs. comfortable	0.10 (−0.60, 0.79)	0.64 (−0.27, 1.55)	0.22 (−0.91, 1.35)
Not enough vs. comfortable	0.37 (−0.25, 0.99)	0.66 (−0.40, 1.73)	0.69 (−0.49, 1.86)
Family history of AD	0.70 (−0.05, 1.45)	1.02 (0.14, 1.90)	1.47 (0.28, 2.66) *
Dementia knowledge	0.04 (−0.07, 0.15)	−0.04 (−0.16, 0.08)	0.20 (0.05, 0.34) **^,†^
Perceived benefit of screening	0.38 (0.21, 0.54) ***	0.37 (0.19, 0.54) ***	0.28 (0.04, 0.52) *
Preventive health behaviors	0.12 (−0.00, 0.24)	0.08 (−0.08, 0.24)	0.06 (−0.12, 0.24)
Perceived susceptibility	0.34 (0.20, 0.48) ***	0.21 (0.04, 0.37) *	0.26 (0.05, 0.46) *
Self-efficacy to get screening	0.24 (0.09, 0.38) **	0.01 (−0.15, 0.17)	0.12 (−0.10, 0.34)
F (*p*-value); R^2^	9.7 (<0.0001); 0.29	5.7 (<0.0001); 0.21	3.3 (0.0002); 0.18

Abbreviations: vs. = versus; HS = high school; AD = Alzheimer’s disease * *p* < 0.05; ** *p*<0.01, *** *p* < 0.001; ^†^ comparison to urban participants significant at *p* < 0.05.
